# Effect of changing threat conditions on police and military commanders’ preferences for urgent and offensive actions: An analysis of decision making at the operational level of war

**DOI:** 10.1080/08995605.2023.2277609

**Published:** 2023-11-03

**Authors:** Jostein Mattingsdal, Bjørn Helge Johnsen, Roar Espevik

**Affiliations:** a Royal Norwegian Naval Academy, Norwegian Defense University College, Oslo, Norway; b Center for Crisis Psychology Faculty of Psychology, University of Bergen, Bergen, Norway; c Leadership and Command & Control Division, Swedish Defense University, Stockholm, Sweden

**Keywords:** Decision making, hybrid attacks, police-military collaborative crisis management, social cognitive theory, force posture, mission urgency

## Abstract

A simulation was conducted to examine the decision making of 102 high-ranking police and military commanders (male/female = 88/12, mean years of employment = 22.15) engaged in a simulated hybrid attack on Norway. Four 2 × 3 repeated-measures ANOVA tests were performed, with two groups (police, military) and three phases (peace, war, and post-conflict) as independent variables. The decision tasks of force posture and mission urgency, along with Subject Matter Expert (SME) ratings of decision-making performance, served as dependent variables. By using social cognitive theory as the theoretical framework, the analysis demonstrated within-group effects indicating how the transition from peace to war caused more offensive postures, higher urgency levels, and increased performance in wartime. Between-group differences were also found, illustrating that police commanders had higher levels of urgency than military commanders in general. Regarding force posture, within-group differences were only found in the post-conflict phase, when police commanders returned to pre-war levels, while military commanders showed less offensive postures than in peacetime. No significant between-group differences were found in decision-making performance. The analysis demonstrated new empirical findings about how crisis management is impacted by change and the backgrounds of those in charge. The findings have implications for designing interagency frameworks that improve police-military interoperability in collaborative efforts.

**What is the public significance of this article?—**This study advances the idea that there are distinct decision-making differences in security crisis defined by the phase in which threats appear, especially how transitioning from peace to war may lead to more offensive and urgent actions by both police and military commanders. It also describes how the commanders' performance was highest in wartime. This highlights how we need to determine ways to improve the police and military's approach to security threats situated below the threshold of war.

## Introduction

The effectiveness of new and emerging security threats has brought revived prominence to debates of how hybrid attacks represent unique difficulties for governmental decision-makers in Western countries (Jensen & Bogart, 2022). In these contexts, hybrid attacks are unusually consequential in the way they combine violent and nonviolent means to exploit the inherent weaknesses of open societies (Weissmann et al., 2021), creating political crises that call for urgent responses by higher levels of government (Dyson & t’Hart, 2013, p. 397). At its core, the cross-sectoral impact of hybrid attacks complicates people’s binary ideas about external and internal security (Bossong & Rhinard, 2021). This transboundary sphere blurs the functional lines between the police and military’s conventional tasks (Lutterbeck, 2004) and has prompted scholars to question the feasibility of current decision-making frameworks (Eriksson & Rhinard, 2009). Their core argument is that even if hybrid attacks impact both the police and military, they are not necessarily managed most effectively through highly sectorized approaches (Speranza, 2020). For this reason, research argues that national governments need an improved understanding of how sectoral vulnerabilities and collective interests enable cohesive approaches to hybrid attacks (Cullen & Reichborn-Kjennerud, 2017, p. 4).

Discussions on decision making in security crises involve descriptions of how threats are perceived by government officials (Herrmann, 2013) and how their interactions can explain a state’s behavior (Redlawsk & Lau, 2013). In this context, a growing number of contributions argue that police-military responsibilities are increasingly contested (Hjellum & Lægreid, 2018) and that the implications of today’s decision-making issues are difficult to predict in future crises (Shepherd & Alistair, 2021). However, their late consequences are exemplified in official reports from recent security crises in the United Kingdom (Murphy, 2006), Israel (Matthews, 2011), Norway (Gjørv et al., 2012), Germany (Fleisher, 2014), and the United States (Hoffman et al., 2015). There is an agreement between the authors that shortcomings in interagency interfaces caused a string of seriously flawed decisions, and all share a preoccupation with improving interagency interoperability by coming to grips with how security crises impact governmental decision-makers.

Against this background, it is remarkable how little knowledge exists about the ways higher levels of government interpret modern security threats (Yarhi-Milo, 2014, p. 23). Many studies illustrate how divergent preferences represent a problem for current perspectives on decision making (Houghton, 2015, p. 290). Even so, scholars argue that we lack empirical insights into the behavior of police and military decision-makers (Shortland et al., 2019, p. 47), even though these agencies have the greatest impact on the outcomes of security crises. Thus, the current study asks the following question: How do changing threat conditions impact the predispositions of police and military commanders toward urgent and offensive actions? The objective was to gain new knowledge about a targeted state’s decision making and to improve the capability of societies to plan, respond to, and recover from hybrid attacks.

## The social cognitive foundation for decisions in hybrid attacks

As the police and military sectors are two distinct domains that intersect in crises (Wither, 2020), the current study used Albert Bandura`s (2011) social cognitive theory (SCT) to analyze how the domain-specific skills of police and military commanders impact the stakes they see and the stance they take. Building on the ways preexisting beliefs are made salient in decisive moments (Gilovich et al., 2002), SCT describes the cognitive processes determining why individuals tend to differ systematically in how they behave. An advantage of SCT is that it explains how uncertain events become informative through self-referent thoughts activated by previous experience from related task domains (Bandura, 1999, p. 181). These anticipatory thoughts motivate individuals by providing them with meaningful, but idiomatic interpretations of their previous experiences’ relevance in the face of the demonstrated efficacy of ongoing actions (Schunk & Usher, 2019). Accordingly, the police and military commanders’ contrasting competencies lead to selective assessments of threats and determine the degrees to which they choose to commit or withdraw resources once new information is considered (Bandura, 1999). Indeed, there are several reasons to expect that such sectoral differences may be even more enhanced in unpredictable and surprising situations (Marchau et al., 2019, p. 28).

Firstly, SCT describes how efficacy beliefs derived from previous experience make decision making easier in both routine and unexpected situations (Bandura, 1999). This is supported by scholars describing how professionals justify assumptions and motivate actions more efficiently than novices (Linou & Kontogiannis, 2004) and that cognitive resources are required in order to know when to surpass standardized procedures in uncertain circumstances (Klein, 2011, p. 28). In this context, SCT explains how individuals exercise control over events by promoting existing abilities or implementing new ones when adapting to change (Bandura, 1986). However, SCT also underlines that there is a marked difference between possessing skills and being able to use them well in dynamic contexts (p. 391). Examples from hybrid attacks include the ability to understand emerging threats, the interpretation of organizational norms, and the strategic use of shared resources to inform and influence actions appropriately.

Secondly, literature on governmental decision making asserts the importance of understanding how preferences are shaped at higher command echelons and the degrees to which they are adjusted over time (Mintz et al., 2021, p. 159). In this context, the research of Halperin et al. (2006) discusses how organizational norms provide powerful perceptual frames that shape decision making in crises. This is supported by research illustrating how professionals prefer methods that have proven effective in day-to-day challenges (Goitein & Bond, 2005, p. 123) and by studies describing how preferences established through personal experience are more robust and more predictive of behavior than weaker preferences (Baumeister & Finkel, 2010, p. 234). However, studies also show that situations involving high stakes tend to challenge people’s preferences (Kunreuther et al., 2002) and that stubborn beliefs can cause vulnerabilities in dynamic conditions (Klein, 2011, p. 5). This effect is described by research showing how self-referent processing of both personal, behavioral, and situational factors tends to produce qualitatively distinct performances across a wide range of situations (Kemeny, 2003, p. 128), including policing (Baldwin et al., 2019) and military operations (Gamble et al., 2018).

Thirdly, when explaining the behavior of government officials, studies show how previous experience is used as reference points that can exacerbate cognitive biases and preclude consideration of options that are outside the scope of their respective organizations (Stein, 2013, p. 371). The specific effects of these self-referent thoughts are discussed by studies claiming that police commanders’ preferences are formed by their enactive and vicarious day-to-day management of immediate operations (Lundgaard, 2021); favoring quick responses (Myhrer, 2015) and avoiding inaction to seize fleeting opportunities (Miner & O’Toole, 2020; Roud, 2021). By contrast, scholars claim that the preferences of military commanders are derived from the deliberate nature of military operations (Vego, 2015), involving delays and adaptive coordination of objectives across multiple tasks and timeframes (Goodwin et al., 2018). According to SCT, these discussions suggest that the current study’s police/military respondents would have different evaluations of their capabilities and what to do with the skills they possess (Bandura, 1997). There are thus good reasons to expect that sector differences readily apparent in the police and military’s unilateral operations will also be evident in collaborative crisis management.

Fourthly, on finding out how threats lead some to withdraw, while others become more risk seeking, Lerner and Keltner (2001) use the heuristics identified by Kahneman and Tversky (1973) to discuss how cognitive appraisals of personal factors influence the activation and maintenance of mobilizing or avoidant behavior. In a social cognitive view, research shows that the most relevant heuristic in uncertain and changing circumstances is probably anchoring-and-adjustment (Cervone & Peake, 1986, p. 492). It refers to how people self-reflectively assess information by comparing it with an initial reference point (i.e., preexisting beliefs and organizational norms) and make adjustments until a plausible estimate is reached (Epley & Gilovich, 2006). As stated by Klein (2011, p. 56), anchoring-and-adjustment often gives correct answers when people must make a decision, but do not know the exact answer (as will be the case when confronting threats that are unexpected relative to the decision-makers’ competencies). Another way to look at anchoring-and-adjustment lies in how SCT explains the emergent nature of people’s thoughts about their ability to perform a task, how this interactive process is based on previous experience determining the number of options considered, and how predictive cues are used selectively to guide behavior (Bandura, 1997). In this context, tasks seen as warranting offensive actions by one could be a defensive task for a second and may even remain an unresolved task for a third.

In sum, the current study regards SCT as a theoretically justified approach for empirical exploration of the decisions police and military commanders must make in security crises. The chief message to be understood from SCT is that decisions are enabled by the ways self-referent thinking predicts future events well enough for commanders to identify courses of action they believe will produce desirable outcomes. In hybrid attack contexts, the vicarious initiation of defensive or offensive operations thus depends not only on the commanders’ efficacy beliefs regarding immediate actions, but also on their inferences about the rules governing how tactical operations are translated into strategic effects. Accordingly, commanders preferring offensive actions will continue, even though this implies conflict escalation and a higher risk of casualties, if they expect persistent offensives to eventually accomplish what they seek. In contrast, the same risks will serve as inhibitors rather than facilitators of offensive actions if they expect that continued offensives will be ineffective (Bandura, 1986, p. 27).

### Hypothesis

The ambiguous nature of hybrid attacks (Weissmann et al., 2021) and the simulation’s bidirectional transitions between peace and war seem to fit the changing settings that SCT predicts will require domain-specific knowledge (Schunk & Usher, 2019) for individuals to assess threats, exercise control, and adjust behavior as the situation calls for it (Grier, 2012; Tsai et al., 2019). In uncertain contexts, scholars claim that individuals tend to redouble their efforts in attempts to gain control (Klein, 2011, p. 228) and that this tendency often leads to more offensive and urgent action in security crises (Feaver, 2009). Considering how SCT describes the functional role that previous experience serves and how crises evoke resolute decision making, the current study first hypothesized (H1a) within-group differences showing that transitioning from peace to war would increase posture and urgency in both the police and military group. Likewise, it was hypothesized (H1b) that both groups’ posture and urgency decisions would return to peacetime levels in the post-conflict phase.

Secondly, the study hypothesized between-group effects in the commanders’ preference for offensive postures and urgent actions. The organizational norms and previous experience of police commanders would have provided excellent insights about the rights and wrongs in civilian crisis management (Bandura, 1997), predisposing them toward taking the initiative and responding quickly to seize fleeting opportunities (Crank, 2015, p. 286). It was thus hypothesized (H2a) that police commanders would demonstrate more offensive and urgent decision making in the peace and post-conflict phase when compared to military commanders. Similarly, the previous experience of military commanders would provide elevated efficacy beliefs in wartime and, as argued by Posen (2014, p. 69), a preference for taking the offensive through counterforce and initiative. As such, it was hypothesized (H2b) that military commanders would demonstrate more offensive actions and greater urgencies in times of war when compared to police commanders.

**Figure 1.**
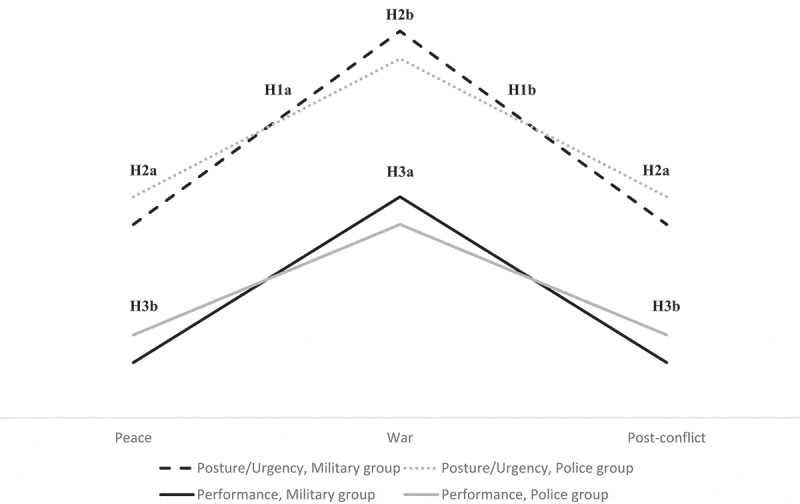
Graphical illustration of the hypotheses developed for investigation.

Thirdly, SCT explains why there is a difference between possessing skills and being able to use them well in change (Bandura, 1986, p. 391), and how the skills of professionals enable them to exercise control and act efficaciously in their respective domains, despite uncertainty (Bandura, 1999, pp. 181–183). Inexperienced commanders could thus demonstrate suboptimal performance, even though they knew what to do, because they questioned the feasibility of unfamiliar, but necessary actions. De Keyser and Woods (1990) demonstrated how this kind of suboptimal performance occurs if individuals are fixated on previous experience and fail to revise assessments. Thus, the commanders’ ability to adapt to change would fail if they relied too heavily on their previous experience. Accordingly, police commanders were expected to have the best insights and perceived high levels of control in times of peace and post-conflict. Similarly, military commanders would be most efficient in wartime conditions. In this context, it was hypothesized (H3a) that military commanders would demonstrate higher decision-making performance than police commanders in wartime. Similarly, police commanders would achieve the highest decision-making performance in peacetime and post conflict (H3b). The hypotheses are illustrated in Figure 1.

## Method

### Respondents

A total of 102 volunteers participated. The inclusion criterion was at least 5 years of active duty in the police or military sector.

The 59 military respondents (53 males and 6 females) were selected from all services and the national joint headquarters (mean age: 44 years, range: 31–58), with 8 to 39 years of active duty and ranks ranging from captain to major-general or equivalent.

The 43 police respondents (34 males and 9 females) were selected from the national police directorate and police districts (mean age: 45 years; range: 29–56), with 8 to 35 years of active duty and ranks ranging from inspector to assistant chief of police.

### Measures

The simulation exercise was conducted at a simulated workstation at the national headquarters with a keyboard and screen facing the respondents. The stimuli were physical handouts (i.e., organizational charts, attribute lists of subordinate forces, legal information, maps, intelligence updates, and policy documents) and digital slides (i.e., mission vignettes and multiple-answer options), with pictures and text projected onto the screen. Computer software iMotions (2022) version 9.1.0.6 controlled the sequence of the slides and recorded all the respondents’ decisions. To allow for realistic dilemmas in the stimuli, the scenario, background documentation, and the 54 mission vignettes were based on high-level scenarios from NATO’s Trident Juncture 2018 exercise (NATO, 2018) and the Occasus setting (Derksen, 2018), in which a fictitious peer-level opponent challenged Norway on a broad arc from Svalbard to the Skagerrak.

Demographic information (age, gender, profession, and years of employment) was collected on the day of the simulation exercise using a printed questionnaire.

### Procedure

Before starting the simulation, the researcher introduced the study’s method and aim of gaining knowledge about factors influencing government officials in hybrid attacks. The respondents were told that their job was to command a national headquarters through a screen-based simulation remotely observed by a researcher. They were informed that the simulation had no time limits that they could withdraw at any point and that once it had started there could be no communication between the respondents and the researcher.

After the respondents had signed an informed consent form, all of the subsequent data was collected electronically in the context of the simulation’s 54 independent missions. The simulation’s three phases (peace, war, and post-conflict) involved equal numbers of missions per phase. The transition from peace to war followed the 18th mission and was initiated by a royal decree announcing a state of war. The re-transition from war to post-conflict followed the 36th mission and was initiated by a reversal of the previously enacted royal decree. All respondents tested the same conditions (i.e., all missions and all phases) in an identical sequence.

### Dependent variables

#### *Force posture* ^1^

**Figure 2.**
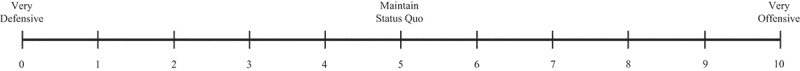
Force posture-scale.

Force posture was measured using a 10-cm Visual Digital Scale (VDS) in each mission (Figure 2). The respondents indicated their force posture-guidance by placing a marker on the VDS. The anchor points were “be very defensive” (i.e., risk of escalation should be avoided) and “be very offensive” (i.e., all necessary coercive techniques may be used). The VDS midpoint indicated that the overall aim was to maintain the status quo, and while escalation should be avoided, the force was allowed to stand its ground. The variable was computed as a sum centimeter over the 18 measurements in each phase.

When conducting assumption checks, the Mauchly’s test of sphericity was not significant (*p* > .05). The Levene’s tests were non-significant in peacetime (F (1,100) = 0.01, *p* = .953); wartime (F (1,100) = 0.19, *p* = .663); and post-conflict (F (1,100) = 0.56, *p* = .457).

#### *Mission urgency* ^2^

**Figure 3.**
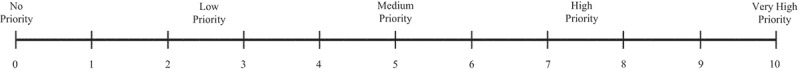
Mission urgency-scale.

Mission urgency was measured by 10-cm VDS in each mission (see Figure 3). The respondents indicated their mission urgency guidance by placing a marker on the VDS. The anchor points were “No priority” (i.e., respond at one’s own convenience) and “very high priority” (i.e., immediate action necessary). The VDS midpoint indicated “respond within the next 24 hours.” The variable was computed as a sum centimeter over the 18 measurements in each phase. The Mauchly’s test of sphericity was significant (ε = .92, *p* = .003), the Huynh–Feldt correction was thus used when calculating the variable’s F-ratios. The Levene's tests were non-significant in peacetime (F (1,100) = 0.01, *p* = .937); wartime (F (1,100) = 2.36, *p* = .127); and post-conflict (F (1,100) = 0.29, *p* = .593).

#### Subject Matter Expert (SME) ratings of decision-making performance

The respondents’ decision-making performance was measured by one police and one military SME who were blind to the experimental setup. The SMEs were selected based on their high levels of academic qualifications and professional knowledge accumulated from more than 30 years of involvement in various security crises. The extent of their competencies thus made them subject matter experts in crisis management.

For each mission per respondent, the SMEs assigned a score to both the force posture and mission urgency decisions as an ordinal variable (0 = low performance), (1 = medium performance), and (2 = high performance). The force posture SME ratings were computed as a sum of the 18 force posture performance scores in each phase. Similarly, the mission urgency SME ratings were computed as a sum of the 18 mission urgency performance scores in each phase.

For the SME-ratings of the force posture-decisions, Mauchly’s test of sphericity was non-significant (*p* > .05). In addition, the Levene's tests were non-significant in peacetime (F (1,100) = 3.38, *p* = .069); wartime (F (1,100) = 2.19, *p* = .142); and post-conflict (F (1,100) = 0.01, *p* = .951).

Conversely, when checking the SME-ratings of mission urgency, Mauchly’s test of sphericity was significant (ε = .92, *p* = .003). The Huynh–Feldt correction was thus used when calculating the variable's F-ratios. The Levene's tests were non-significant in peacetime (F (1,100) = 3.39, *p* = .069) and wartime (F (1,100) = 1.34, *p* = .249). However, in the post-conflict phase the Levene's test was significant (F (1,100) = 6.16, *p* = .015). To this end, a robust ANOVA test (Field & Wilcox, 2017) was conducted to investigate whether the groups' mission urgency decision-making performance differed in the post-conflict phase.

The composite scores of both SMEs were computed to calculate the intraclass correlation coefficient (ICC) on both variables. The force posture expert ratings’ interrater reliability showed a very good ICC of .800 (*p* < .001). The mission urgency expert ratings’ interrater reliability showed an acceptable ICC of .715 (*p* < .001).

### Independent variables

The independent variables were Sector (police/military) and Phase (peace/war/post-conflict).

### Statistics

The data were analyzed in statistics software Jamovi (2023) version 2.3.26 using four 2 × 3 repeated measures analysis of variance (ANOVA) tests with subsequent post-hoc Tukey tests. The alpha level of significance was set to 0.05 (5%).

The first ANOVA tested the commanders’ force posture decisions, while the second looked at mission urgency. The purpose was to determine how far the independent variables were major sources of decision-making variability. The third ANOVA tested the SME ratings of force posture decision-making performance, while the fourth considered mission urgency decision-making performance. Our interest was to compare the commanders’ relative performance across the phases, and whether it changed as the scenario transformed from peace to war, to post-conflict. Partial Eta Squared (η_p_2) were calculated to measure the proportion of variances attributable to the effect under consideration. The reference values for effect sizes for Partial Eta Squared are as follows: Small effect (S) = .01; medium effect (M) = .06; and large effect (L) = .14 (Maher et al., 2013). There were no missing data.

## Results

Tables 1–4 present the means, standard deviations, and Pearson r correlations for the variables: force posture, mission urgency, force posture decision-making performance, and mission urgency decision-making performance. Correlations are reported with the degrees-of-freedom number 100.

**Table 1.** Correlation matrix, force posture.

	Peacetime Posture	Wartime Posture	M	SD
Peacetime Posture	–	–	125.74	15.64
Wartime Posture	.59***	–	145.17	13.82
Post-Conflict Posture	.70***	.63***	121.10	20.02

***p > .001, M = Mean, SD = Standard deviation.

**Table 2.** Correlation matrix, mission urgency.

	Peacetime Urgency	Wartime Urgency	M	SD
Peacetime Urgency	–	–	129.22	18.66
Wartime Urgency	.74***	–	146.03	18.83
Post-Conflict Urgency	.61***	.75***	130.95	20.50

***p > .001, M = Mean, SD = Standard deviation.

**Table 3.** Correlation matrix, decision-making performance, force posture.

	Peacetime Performance Posture	Wartime Performance Posture	M	SD
Peacetime Performance Posture	–	–	24.50	3.07
Wartime Performance Posture	.27**	–	27.26	2.81
Post-Conflict Performance Posture	.30**	.13	25.71	2.89

**p > .01, M = Mean, SD = Standard deviation.

**Table 4.** Correlation matrix, decision-making performance, mission urgency.

	Peacetime Performance Urgency	Wartime Performance Urgency	M	SD
Peacetime Performance Urgency	–	–	24.54	3.55
Wartime Performance Urgency	.35***	–	25.27	2.31
Post-Conflict Performance Urgency	.41***	.40***	23.52	4.81

***p > .001, M = Mean, SD = Standard deviation.

Although the descriptive statistics and correlation coefficients illustrate the associations between variables related to the hypotheses, four ANOVA tests were necessary to determine the main effects and interaction effects of the variables in the police and military group.

### Force posture

The analysis of the force posture data showed a main effect of Sector, F(1, 100) = 5.84, *p* = .017, η_p_^2^ = .06 (M), with police commanders demonstrating the most offensive posture levels in general. A main effect of Phase was also found, F(2, 200) = 152.17, *p* < .001, η_p_^2^ = .60 (L). H1a was supported by a post-hoc test indicating more offensive levels of force posture in war relative to both in peace (*p* < .001) and the post-conflict phase (*p* < .001). Contrary to H1b, the results indicated that the commanders’ force posture levels were more offensive in peacetime than in post conflict (*p* = .002). The analysis also showed an interaction of Phase × Sector, F(2, 200) = 4.27, *p* = .015, η_p_^2^ = .04 (S). A post-hoc Tukey test revealed further support for H1a by describing how military commanders preferred higher levels of force posture in war than in peace (*p* < .001) and the post-conflict phase (*p* < .001). Similarly, the Tukey test showed that police commanders had increased levels of force posture in war relative to in peace (*p* < .001) and the post-conflict phase (*p* < .001).

**Figure 4.**
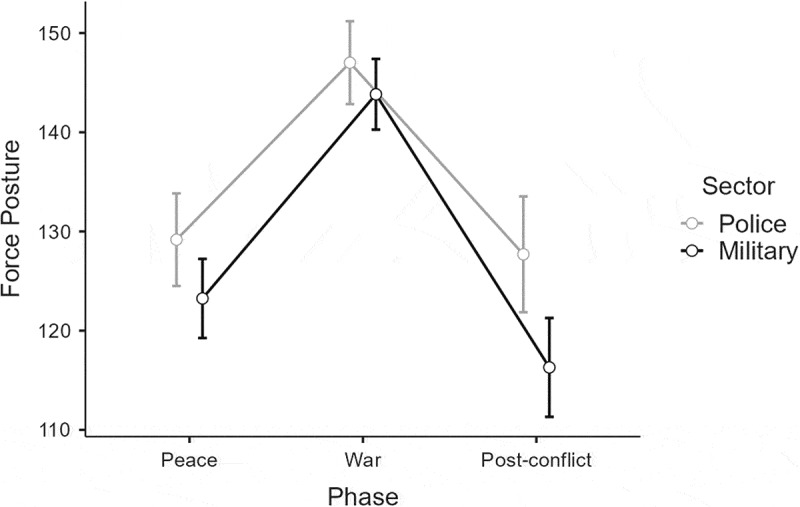
The commanders’ force-posture levels in the three phases.

Note: Error bars indicate 0.95 confidence intervals.

Additionally, the Tukey test showed support for H1b in the police group, as their force posture levels did not achieve significance in comparing post conflict and peacetime. Interestingly, H1b was contradicted by the military group. The military commanders’ posture levels were lower in the post-conflict phase than in peace (*p* = .004). The Tukey test also showed support for H2a by the ways in which police commanders demonstrated higher levels of force posture than military commanders in the post-conflict phase (*p* = .045), as shown in Figure 4.

### Mission urgency

The second analysis explored the commanders’ mission urgency decisions. The results showed a main effect of Sector, F(1, 100) = 24.42, *p* < .001, η_p_^2^ = .20 (L), with police commanders demonstrating the highest mission urgency levels. A Huynh–Feldt corrected main effect of Phase was found, F(2, 200) = 73.47, *p* < .001, η_p_^2^ = .42 (L). H1a was supported by a post-hoc test showing higher levels of urgency in war than in both peace (*p* < .001) and the post-conflict phase (*p* < .001). H1b was also supported, as the comparison of peace and post-conflict did not reach significance. The interaction of Phase × Sector was non-significant, but as explained by Wilcox (1987, p. 36), multiple-comparison procedures can be used when a hypothesis of differences exists, regardless of whether the F-test is significant.

**Figure 5.**
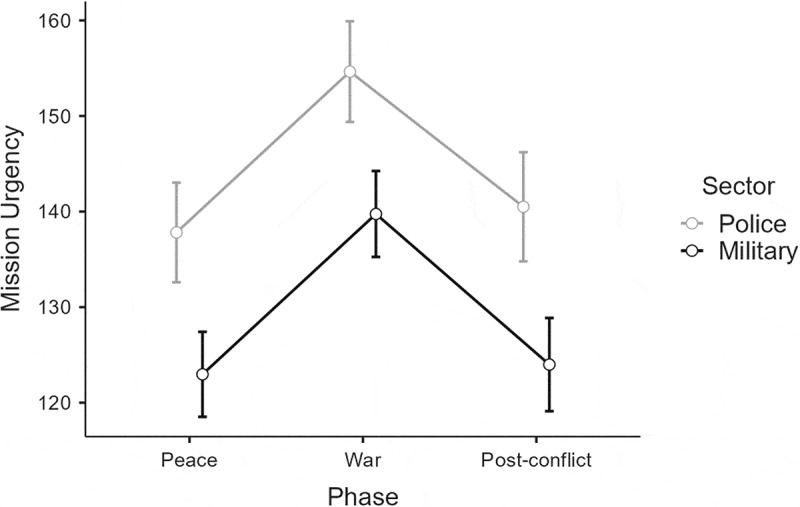
The commanders’ mission-urgency levels in the three phases.

Note: Error bars indicate 0.95 confidence intervals.

Considering the current study’s hypothesis concerning between-group differences in the transitions between peace and war, a post-hoc Tukey test was thus justified. It elaborated support for H1a by describing how both police and military commanders demonstrated higher levels of urgency in war when compared to peace (*p* < .001) and the post-conflict phase (*p* < .001). The Tukey test also supported H2a by showing that police commanders demonstrated higher levels of urgency than military commanders in peace (*p* < .001) and in the post-conflict phase (*p* < .001). Contrary to H2b, the results showed that police commanders had higher levels of urgency than military commanders in war (*p* < .001), as shown in Figure 5.

### Force posture decision-making performance

**Figure 6.**
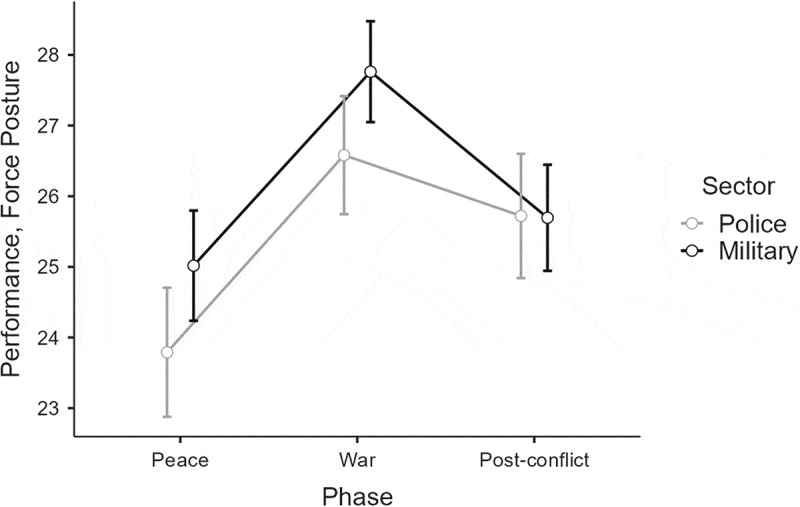
SME ratings of the commanders’ force-posture decision-making performance in the three phases.

Note. Error bars indicate 0.95 confidence intervals.

The third analysis explored the SME ratings of the police and military commanders’ force posture decisions (see Figure 6). The results showed a main effect of Phase, F(2, 200) = 29.47, *p* < .001, η_p_^2^ = .23 (L). A post-hoc test indicated how the commanders’ wartime force-posture decisions achieved higher ratings than in peace (*p < *.001) and in the post-conflict phase (*p* < .001). On comparing the post-conflict phase with peace, the ratings were lowest in peace (*p* < .001). The analysis also showed a borderline significant main effect of Sector, F(1, 100) = 3.84, *p* = .053, η_p_^2^ = .04 (S), with military commanders achieving slightly higher ratings than police commanders. A significant interaction of Phase × Sector was not found, but since our hypothesis implied sector differences across the phases, a post-hoc Tukey test was conducted.

The Tukey test demonstrated that military commanders achieved higher ratings in war than in both peace (*p* < .001) and the post-conflict phase (*p* < .001). The SME ratings of the police commanders’ decisions in war were higher than in peacetime (*p* < .001), but not in the post-conflict phase. The results also showed how police commanders achieved lower ratings in peace than in the post-conflict phase (*p* = .006). Interestingly, none of the between-group comparisons reached significance. As such, H3a was not supported; military commanders did not achieve higher decision-making performance than police commanders in wartime. Similarly, H3b was also not supported; police commanders did not achieve higher decision-making performance than military commanders in the peace and post-conflict phase.

### Mission urgency decision-making performance

**Figure 7.**
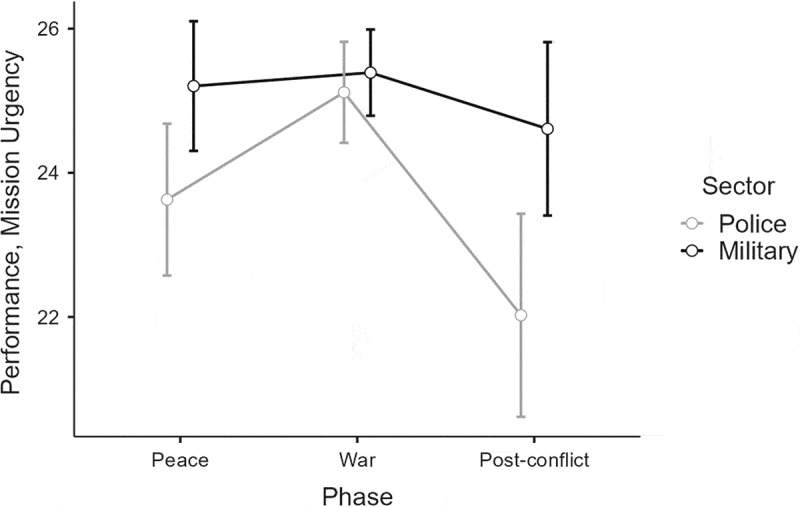
SME ratings of the commanders’ mission-urgency decision-making performance in the three phases.

Note. Error bars indicate 0.95 confidence intervals.

The fourth analysis explored the SME ratings of the police and military commanders’ mission urgency decisions (see Figure 7). The results showed a main effect of Sector, F(1, 100) = 7.46, *p* = .007, η_p_^2^ = .07 (M), indicating how the military commanders’ urgency decisions generally achieved higher SME ratings across the simulation. As discussed in the methods-section, the *p*-value should be considered unreliable due to the significant Levene's test, but the partial eta squared can still be interpreted as it neither requires normality or homogeneity (Levine & Hullett, 2010).

A Huynh–Feldt corrected main effect of Phase was also found, F(2, 200) = 10.84, *p* < .001, η_p_^2^ = .10 (M). A post-hoc test indicated how the police and military commanders’ mission-urgency decisions achieved higher ratings in war than in peace (*p* = .036) and the post-conflict phase (*p* < .001). The analysis also showed an interaction of Phase × Sector, F(2, 200) = 3.86, *p* = .023, η_p_^2^ = .04 (S). A post-hoc Tukey test showed a borderline significant interaction *(p* = .061) suggesting that the police commanders achieved higher ratings in war than in peacetime. Additionally, the Tukey test indicated that police commanders achieved lower ratings in the post-conflict phase than in war (*p* < .001).

The between-group comparisons showed no support for either H3a or H3b. However, a borderline significant interaction *(p* = .071) supported by a significant (*p* = .023) robust ANOVA test suggests that the military commanders achieved higher SME-ratings than the police in the post-conflict phase. Although low in magnitude, this result could be interpreted as partly contradicting H3b.

## Discussion

The current study's aim was to gain new knowledge about the actions of police and military commanders engaged in decision-making regarding force posture and mission urgency in the context of hybrid warfare. The following hypotheses were raised from the above stated problems. H1a: Transitioning from peace to war will result in increased levels of force posture and mission urgency in both the police and military groups. H1b: The force posture and mission urgency decisions of both groups will return to peacetime levels in the post-conflict phase. H2a: Police commanders will demonstrate more offensive and urgent decision making in the peace and post-conflict phase when compared to military commanders. H2b: Military commanders will demonstrate more offensive actions and greater urgencies in times of war when compared to police commanders. H3a: Military commanders will demonstrate higher decision-making performance than police commanders in wartime. H3b: Police commanders will demonstrate higher decision-making performance than military commanders in the peace and post-conflict phase.

The analysis showed that the phase transition from peace to war made both police and military commanders demonstrate higher levels of force posture and mission-urgency decision making, which supported H1a. The strength of the relationship between escalation and decision making can be considered to have practical significance, because the main effects’ size was large for both posture (η_p_^2^ = .60) and urgency (η_p_^2^ = .42). In addition, given that all respondents were high-ranking commanders, and the scenario closely resembled real-life security challenges, this finding strongly indicates that crisis escalation not only increased the commanders’ preferences toward offensive action, but also their sense of urgency. It shows how a contextual shift in reference point from peace to war fundamentally altered the decision making of police and military commanders.

One possible explanation is that the commanders interpreted the escalatory transition to war as inconsistent with strategic aims, triggering cognitive discrepancies between preexisting beliefs and current performance. SCT explains how this will motivate behavior based on anticipatory estimations of what is required to resolve the perceived discrepancy (Bandura, 1999, p. 176). This self-mobilizing mechanism may involve evoking directional motivations toward expending more resources on attempts to regain control. In this view, the present finding strongly illustrates how this self-referent mechanism came into play, as both the police and military commanders were more likely to act aggressively after transitioning to war, consequently raising the probability of further escalation. Furthermore, this elaborates studies showing that government officials are influenced by the same psychological processes as novices (Sheffer et al., 2018) and supports scholars who argue that urgent and offensive action will be favored over holding back when crises escalate and war is seen as inevitable (Jervis, 2017, p. 222).

In contrast, the transition from war to post-conflict resulted in differential decision making. However, the between-group findings cannot be considered very strong, because the interaction effects’ strength was low for posture (η_p_^2^ = .04) and very low for urgency (η_p_^2^ = <.01). Even so, the analysis showed that the police commanders’ decisions regarding both force posture and mission urgency returned to peacetime levels in the post-conflict phase, which supported H1b. This finding may reflect that the police commanders perceived few disparities between the peace and post-conflict phases. If so, SCT explains that the police commanders would mobilize less effort toward engaging new concepts and, as a result, would be more resistant to change (Bandura, 1999). Interestingly, military commanders showed the same tendency regarding mission urgency but not force posture. Here, the data described how military commanders were more defensive in post-conflict than in the initial peace condition, which contradicted H1b. This finding may indicate that events which the police interpreted as warranting offensive action in de-escalating scenarios were seen as unnecessary use of force by the military. As such, the finding shows how police commanders renormalized their anticipatory estimations faster than the military commanders, when recovering from security crises. This helps explain why some commanders will strive for a return to familiar conditions, while others will move beyond and think on the margins in crises. The realization is then that these mechanisms need to be understood to enable a certain degree of improvisation and flexibility in collaborative efforts.

The mixed support for H1b supports previous research showing how change often results in differential responses by the same individual, depending on the regulatory strategies they choose to interpret new events (Bandura, 1999). In this context, a possible explanation for why police commanders returned to peacetime levels, while the military commanders were more defensive in the post-conflict phase than in peacetime, is that the commanders’ cognitive readiness (Grier, 2012) and preexisting beliefs had varying levels of robustness. This explains how the subsequent selective interpretations of events would have provided them with a mix of pros and cons that motivated behavior in divergent directions.

The police commanders’ tendency to renormalize their reference point illustrates the psychological necessity of making assumptions in line with previous experience (Elstein et al., 1990). This may have undermined the police commanders’ willingness to choose untried and untested courses of action in de-escalating contexts. In contrast, military commanders seemed less fixated on peacetime reference points when making decisions in the post-conflict phase. Although the result points to the potential effect of changing threat conditions, critics argue that biased assessments due to change are much rarer than commonly supposed (Guess & Coppock, 2020) and that shifts from moderate to more extreme positions are an infrequent outcome when people are exposed to uncertainty (Kuhn & Lao, 1996). Even so, the current findings illustrate important sector differences in the ways commanders made differential tradeoffs between adaptation and pursuing preexisting beliefs in de-escalating circumstances. To this end, our result reflects the general coordination problems observed in Norwegian crisis preparedness (Rykkja & Lægreid, 2014) and how the present sector-based organization may lead to an increase in sunk costs to organizational norms, further raising the threshold for implementing more transformational solutions (Marchau et al., 2019, p. 503).

It is possible that the military commanders’ formal training in peacekeeping and previous experience from international operations is the explanation for why they adapted differently than the police commanders in the post-conflict phase. As explained by Klein (2017), the military commanders’ domain-specific skills would have given them a greater ability than police commanders to differentiate the peace and post-conflict phase, either through previous experience or through deliberate calculations. When this line of reasoning is applied to the police commanders’ undifferentiated decision making across the same conditions, it is possible that it represents how policing in post-conflict scenarios was interpreted according to the police sector’s peacetime norms. As such, the conditions of post conflict seem to have been below the police commanders’ threshold for adaptation.

From these findings, it follows how reliance on preexisting beliefs can result in differential decision making that complicates the implementation of alternative concepts even amid ongoing crises. Thus, the analysis reveals a somewhat surprising pattern. The transition from peace to war clearly generated more offensive actions and greater urgency for everyone, while the transition from war to post-conflict created differential decision making. From a social cognitive perspective, this shows that even if justifications for change are evident, there may be insufficient feedback to motivate a search for alternative actions, as might be the case for the police commanders. On the other hand, SCT (McCormick, 2001) also explains how the challenges of uncertainty can be resolved if events are perceived as supporting new strategies, as might be the case for the military commanders. This finding elaborates previous studies describing the decision-making difficulties presented by modern conflicts (Shortland et al., 2019) and how transboundary threats complicate cross-sectoral collaboration (Sarapuu et al., 2014).

The study’s other hypotheses also warrant discussion. For example, the Sector variable’s substantial effect size (large for urgency; η_p_^2^ = .20 and medium for posture; η_p_^2^ = .06) constitutes a reason to reflect on its real-world implications. The data showed that police commanders demonstrated more offensive postures, as well as greater urgencies, than military commanders in the peace and post-conflict phases, which supported H2a. On the one hand, this indicates how the police commanders were optimistic about the relevance of their previous experience in both peacetime and post conflict. On the other hand, it may show that military commanders considered that holding peacetime events as reference points for decision making in post conflict would yield less utility than adopting more restrictive approaches. As this effect was not found among the police commanders, this finding shows how the transition from war to post-conflict impacted police and military commanders differently. Moreover, it supports the ways SCT explains that exercising control in unfamiliar situations is not just a matter of gaining predictive knowledge, but of gaining the self-assurance necessary to act decisively (Bandura, 1997). The commanders’ efficacy beliefs may thus be useful to better understand the demonstrated sector differences.

Interestingly, while the police commanders returned fully to peacetime posture levels in the post-conflict phase, the military commanders’ force postures were significantly lower across the same condition. This appears to be an adaptability type of effect (i.e., events in post-conflict seem to have been below a response threshold according to previous military experience). As shown by Dettaff et al. (2020, p. 88), previous experience and organizational norms function as subjective criteria that must be exceeded if judgments are to evolve into actions. The post-conflict condition seems to have represented a sub-threshold stimulus for the military commanders, resulting in behavioral adjustments dissimilar to those previously conducted in peacetime and war. In parallel, military commanders did not demonstrate more offensive postures than police commanders in wartime, which did not support H2b. The data also showed that police commanders had higher levels of urgency than military commanders in war, which was contrary to H2b.

The mixed within-group effects seem to illustrate how police and military commanders felt dissimilar pressures to be offensive and act urgently across the simulation. The twist of this finding reinforces SCT’s prediction that the impact of change on behavior depends on the decision-makers’ self-referent retrospective reasoning and forethoughts about the relationship between ongoing events and future actions (Bandura, 1999).

The implications of these psychological findings are several. First, they explain why behavioral outcomes in security crises may be as much the product of the ways decision-makers think as a result of an event’s objective incentives (Jervis, 2017). Secondly, they elaborate why the police and military’s operational environment has both discrepancies and commonalities (Penney et al., 2022) and why it is premature to conclude that sector differences will correspond across change. Thirdly, the results seem to expand the arguments of scholars claiming that police commanders are predisposed toward rapid responses (Crank, 2015; Myhrer, 2015). To the extent that this makes withdrawal difficult, police commanders may be less aware of how urgent actions entail a high chance of escalation and that holding back can be advantageous for crisis stability in hybrid attacks. None of this is to say that military commanders are more aware of change and less likely to engage in unwarranted actions. As shown by Vallée-Tourangeau et al. (2011), falling back on previous experience can allow decision-makers to make feasible decisions, but in other instances can make it difficult to think of new ways of solving problems. In this context, the differential outcomes between police and military commanders shown in the present study expand the area of naturalistic decision making (Mosier et al., 2018) and support SCT’s predictions that self-referent thinking is a critical ability responsible for regulating decision-making behavior in organizational settings (Stajkovic & Sergent, 2019, p. 10). Thus, a more detailed analysis of the ways these cognitive tendencies are manifested in other public sectors may potentially be important, and further empirical studies should investigate this in detail.

The study’s final findings of note regard the SME ratings of the commanders’ decision-making performance. Firstly, the analysis demonstrated that decision-making performance was highest in wartime. This is an important finding that is backed by a strong effect size for both posture (η_p_^2^ = .23) and urgency (η_p_^2^ = .10). It expands recent studies arguing how hybrid attacks taking place below the threshold of war complicate decision making (Cullen & Reichborn-Kjennerud, 2017, p. 31). Interestingly, previous studies have both demonstrated (Levi & Tetlock, 1980) and contradicted (Suedfeld & Bluck, 1988) that decision-making performance tends to decline in war.

Secondly, no significant differences were found in the police and military commanders’ decision-making performance in wartime, which did not support H3a. Similarly, no significant differences were found when analyzing the commanders’ decision-making performance in peace and post conflict, which did not support H3b. However, the expert ratings of mission urgency showed a borderline significant effect *(p* = .071) with a medium effect size (η_p_^2^ = .07) that were supported by a significant robust ANOVA test *(p* = .023) in the post-conflict condition. Although not very large, the result suggests that police commanders somehow failed to revisit their assessments adequately when the scenario changed. This complements the above finding about divergent urgency levels in post conflict (H2a) and illustrates how the self-referent anchoring mechanisms of police and military commanders created slightly divergent behavioral adjustments after transitioning to post-conflict. Even so, one should be careful to draw any conclusions about the relatively cognitive readiness of the commanders. The results cannot support the idea that either the police or the military should unilaterally lead the way in operations to counter hybrid warfare. However, they support previous research asserting the importance of interagency approaches (Bynander & Nohrstedt, 2019) and advance our understanding of hybrid warfare by describing some of the unique decision-making challenges it creates in collaborative crisis management.

Thirdly, the qualitative differences illustrated by the expert ratings support research demonstrating how the ability to regulate behavior in response to changing conditions is an important ability (Joseph & Ocasio, 2012; Laureiro‐Martínez & Brusoni, 2018) to manage the cognitive shifts (Foldy et al., 2008) needed for efficient exploration of options in uncertain circumstances (Marcel et al., 2011). As argued by Klein (2011, p. 5), this implies that decision-makers must recognize when preexisting beliefs are valid, while maintaining the ability to acknowledge when improvisation is needed. The current analysis found that the commanders’ decision making was not random but was based on anticipatory thinking derived from domain-specific occupational knowledge. We believe that this provides powerful evidence that the commanders’ actions were largely a product of the self-referent mechanisms described by SCT (Bandura, 1986).

### Limitations

The present empirical analysis of decision making in hybrid attacks may have important implications for future crisis management efforts but is not without limitations. Although both groups showed increased levels of offensiveness in wartime, the analysis did not indicate that the transition from peace to war impacted the police and military commanders differently (i.e., the analysis did not indicate that the parameters increased unequally in the transition to war). As such, the severity of war seems to have activated a set of shared beliefs that are common to both the police and military in wartime conditions. Another possible reason for the lack of findings in the war phase is that the choice of statistical tests was suboptimal, or the simulation may have been insufficient to identify sector differences. Alternatively, the force posture measurement may not have captured actual predisposition toward offensive actions in wartime. For example, the study measured the commanders’ force posture decisions on a defensive-offensive scale, which says little about the actual use of force. Future research should thus consider investigating the corresponding coordinating instructions (i.e., the order of actions, planned formations, and control measures that pertained to each mission).

Regarding the SME ratings, we developed a scale to assess the commanders’ decision-making performance. However, we acknowledge that the scale did not follow a traditional construction process and that the analysis cannot explain the lack of differential group effects, although one finding was borderline significant, with a medium effect size. Even though the scale allowed the commanders to adjust their decisions in ways that are relevant when conducting operations, the lack of support for H3a and H3b could suggest that the scale had too low resolution (i.e., only three levels), or that our choice of task elements should have included other elements (i.e., the information aspect). This could have resulted in further explanations but was excluded due to our choice of statistical tests and study design. Based on these findings, we recommend future studies to maintain the current decision elements (force posture and mission urgency) but suggest constructing a scale in accordance with validated psychological-scale concepts.

Although the current study measured the commanders’ regulation of force posture and mission urgency, it did not fully assess the specific variables related to SCT (i.e., self-efficacy). As such, continued investigation of the self-regulatory mechanisms proposed by SCT would add to our understanding of the nuances between the present simulation and previous research of the impact of self-referent thinking on decision making. As asserted by recent studies, future research should investigate how multiple individuals (Gore et al., 2018) and collective efficacy (Krammer et al., 2018) can explain the decision making of command teams across changing conditions.

## Conclusion

This study’s main result describes how both police and military commanders demonstrated more offensive postures and higher levels of urgency in wartime than in otherwise similar tasks in times of peace and post-conflict. Furthermore, the analysis showed significant-sector differences in the ways police and military commanders adjusted their preferences to change, and as a result, how their decision-making efficacy varied. It not only demonstrates the varying robustness of the commanders’ preexisting beliefs but also that sectoral affiliation had a strong impact on the ways decisions were made. These findings clearly illustrate how and why some operational solutions proved more psychologically appealing than others. In this context, the current study expands previous research on the behavior of professionals (Hoffman et al., 2013; Schraagen et al., 2008; Shortland et al., 2019) and sheds light on how the interactions of sectors with distinct types of preferences may account for decision-making outcomes that impair performance.

Combined with the theoretically justified predictions of SCT (Bandura, 2018), the analysis points to some important challenges and helps understand why the psychological effects of phase transitions are central for understanding new and emerging security threats. We believe these findings can help improve the police-military dialogue and make a difference in improving interoperability and reducing risk in collaborative efforts. Extending this research to other governmental sectors would be particularly valuable because, unlike the hybrid attacks explored here, most crises do not entail security threats. However, these crises (i.e., natural disasters, financial crises, organizational crises) are far more prevalent and equally consequential for the individuals and organizations involved.

## Data Availability

All data, analysis code, and research materials are made available to experts in the field with explicit permission from the Norwegian Defense University College by contacting the authors at, jomattingsdal@mil.no.

## Notes

1. Force posture involved the commanders’ intent regarding the use of force in operations. It was not a limitation but provided subordinate forces with an understanding of the expected readiness of force elements, the type of methods that were suitable and how much risk the commander accepted. For example, defensive postures indicated that the goal was to de-escalate the situation. Conversely, offensive postures meant that the force could take the initiative and that escalation was acceptable.

2. Mission urgency involved the commanders’ intent regarding the expected time from when a force received orders to conduct a given mission to the time movement was initiated. It included the time for planning, order briefings, and necessary preparations to familiarize capabilities, connect commands, and integrate combat support.
